# The Novel Diketopiperazine Derivative, Compound 5-3, Selectively Inhibited the Proliferation of FLT3-ITD Mutant Acute Myeloid Leukemia (AML) Cells

**DOI:** 10.3390/md23070289

**Published:** 2025-07-16

**Authors:** Shijie Bi, Yating Cao, Shiyuan Fang, Yanyan Chu, Zixuan Zhang, Meng Li, Rilei Yu, Jinbo Yang, Yu Tang, Peiju Qiu

**Affiliations:** 1 Key Laboratory of Marine Drugs, Chinese Ministry of Education, School of Medicine and Pharmacy, Ocean University of China, 5 Yushan Rd, Qingdao 266003, China; bshijie@yeah.net (S.B.); 15763381936@163.com (Y.C.); fangshiyuan@163.com (S.F.); zzx7212@stu.ouc.edu.cn (Z.Z.); 18839127899@163.com (M.L.); ryu@ouc.edu.cn (R.Y.); yangjb@ouc.edu.cn (J.Y.); 2 Marine Biomedical Research Institute of Qiangdao, 23 Hongkong East Rd, Qingdao 266003, China

**Keywords:** acute myeloid leukemia, FLT3-ITD mutation, diketopiperazine, tubulin

## Abstract

The internal tandem duplication mutation of FMS-like tyrosine kinase 3 (FLT3-ITD) is associated with high recurrence and mortality rates in acute myeloid leukemia (AML), making it a critical target for anti-AML therapies. Plinabulin is a diketopiperazines derivative that exhibits extensive anti-cancer potency by targeting β-tubulin. We designed and synthesized a novel FLT3 inhibitor, namely **5-3**, based on the structure of plinabulin and evaluated its effect on FLT3-ITD mutant AML cells. The results indicated that **5-3** potently and selectively inhibits the growth of mutant FLT3-expressingleukemia cells, and had no effect on FLT3 wide-type cancer cells, suggesting the antiproliferative activity of **5-3** depends highly on FLT3-ITD expression. Mechanically, **5-3** significantly suppressed the phosphorylation of FLT3 signaling pathway, including STAT5, Erk and Akt. Moreover, the efficiency of compound **5-3** is not associated with Plinabulin’s typical target, β-tubulin. In conclusion, the study identified diketopiperazine derivative as a novel FLT3-ITD selective inhibitor. These results demonstrated that **5-3** might be a drug candidate for the treatment of FLT3-ITD-positive AML.

## 1. Introduction

Mutations of the FMS-like tyrosine kinase 3 (FLT3) gene occur in approximately 30% of all AML cases, with the internal tandem duplication (ITD) representing the most common type of FLT3 mutation (FLT3-ITD; approximately 25% of all AML cases) [1,2]. The FLT3-ITD mutation serves as a critical driver mutation in the pathogenesis and progression of acute myeloid leukemia (AML) and represents a key focus for the development of anti-AML targeted therapies. Since 2017, three FLT3 inhibitors (Midostaurin, Gilteritinib, and Quizartinib) have been approved for the treatment of patients with FLT3-mutated AML. Furthermore, Clifutinib and XY0206 are currently in phase III clinical trials [3,4].

Based on their binding modes with FLT3, FLT3 inhibitors can be categorized into Type I and Type II. Type I FLT3 inhibitors bind to both the active and inactive conformations within the ATP-binding region of the kinase. Given the structural similarity of the ATP-binding regions across most kinases, many Type I inhibitors exhibit activity against multiple kinases. In contrast, Type II inhibitors bind exclusively to the inactive conformation. Their action site is located in the hydrophobic region adjacent to the ATP-binding region. Due to the lower conservation of the hydrophobic region compared to the ATP-binding region, Type II FLT3 inhibitors generally demonstrate higher selectivity. Representative examples of Type II FLT3 inhibitors include Quizartinib (AC220), Sorafenib, and Crenolanib [5,6].

Despite the significant clinical efficacy demonstrated by the three marketed FLT3 inhibitors, as with other kinase inhibitors, resistance frequently emerges, rendering them ineffective over time. For example, resistance to Gilteritinib typically develops after 6–7 months of monotherapy [7], AC220 resistance occurs after approximately 4 months of monotherapy [8], and Midostaurin resistance arises within several weeks to several months of use [9]. The development of resistance markedly shortens the clinical benefit duration of FLT3 inhibitors and ultimately contributes to disease recurrence. There is an urgent need to develop novel FLT3 inhibitors.

Diketopiperazines (DKPs) are a class of essential natural products primarily produced by marine microorganisms. These compounds belong to the category of dipeptides formed via the condensation of two α-amino acids through peptide bonds. The framework consists of a six-membered ring with two hydrogen bond donors and two hydrogen bond acceptors. The stable six-membered ring structure of dihydrooxazine compounds renders them highly significant in pharmacology. They can act as DNA binders, microtubule depolymerizers, rho inhibitors, quorum sensing modulators, antifungals, oxytocin antagonists, PDE5 inhibitors, and neuroprotective agents. Among them, the most important one is, plinabulin (NPI-2358), which was isolated from the marine fungus Aspergillus ustus, has been demonstrated to be an effective tubulin depolymerizing agent and is phase 3clinical stage for treating non-small cell lung cancer [10,11,12,13,14,15,16,17,18].

Previously, we reported a series of novel plinabulin derivatives that exhibited broad-spectrum antitumor activity against colon cancer, lung cancer, and pancreatic cancer by targeting β-tubulin [19]. However, these compounds lacked selectivity for killing FLT3-ITD mutant AML cells. Based on the essential binding mode of Type II FLT3 inhibitors with FLT3, we designed and synthesized a novel FLT3 inhibitor, **5-3**, which derived from the unique structure of plinabulin and exhibited selective inhibition on FLT3-ITD mutant AML cells. In addition, compound **5-3** exhibited activity beyond the tubulin target, suggesting a novel mechanism of diketopiperazines in killing cancer cells.

## 2. Results

### 2.1. **5-3** Potently and Selectively Inhibits Thegrowth of Mutant FLT3-Expressingleukemia Cells in Vitro

**Figure 1:**
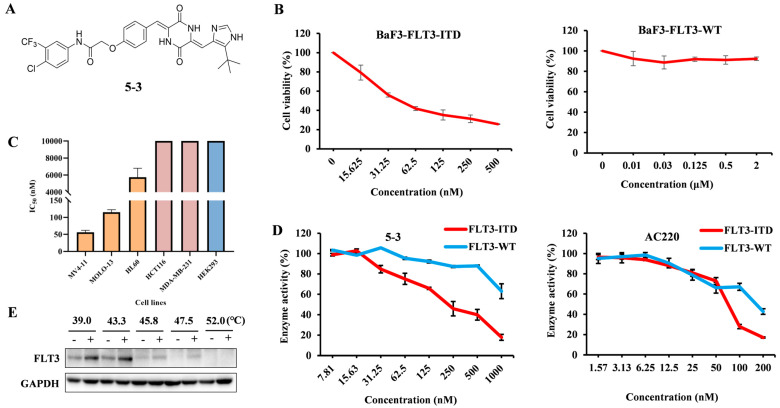
The proliferation of FLT3-ITD mutant cells was selectively inhibited by ** 5-3**. (**A**) The chemical structure of **5-3**. (**B**) BaF3 transfectants stably expressing wild-type FLT3 or mutated FLT3s were treated with compound **5-3** for 48 h. After treatment, cell viability was assessed by MTT assay. (**C**) The IC_50_ of **5-3** on multiple cancer cells. (**D**) The inhibition curve of **5-3** on FLT3-ITD and FLT3-WT enzyme. (**E**) CETSA assays verified the interaction of **5-3** with FLT3 protein in MV4-11 cells.

As illustrated in Figure 1A,B, the IC_50_ of **5-3** to suppress the growth of FLT3-ITD and FLT3-WT overexpressing BaF3 is 24.2 nM and >2000 nM, respectively, suggesting that **5-3** selective inhibits the growth of FLT-ITD mutant cells. Moreover, **5-3** potently inhibited the proliferation of MV4-11 (IC_50_ = 51.93 nM, 100% FLT3-ITD mutation) and showed greater efficiency than MOLM13 cells (IC_50_ = 115.35 nM, FLT3-ITD mutation and FLT3-WT). The IC_50_ in FLT3 wide-type HL60 cells and other cell types were more than 6000 nM (Figure 1C). These data show that compound **5-3** has superior specificity for AML cells with FLT3-ITD mutation compared to other tumor cells and confirm that the antiproliferative activity of **5-3** depends highly on FLT3-ITD expression.

The kinase assay results revealed that **5-3** potently inhibited kinase activities of wild-type FLT3 and FLT3-ITD with IC_50_ values of >1000 nM and 188 nM, respectively. The IC_50_ values of AC220 for wild-type FLT3 and FLT3-ITD is 171.5 nM and 68.05 nM, respectively. The ratio of wild-type FLT3 to FLT3-ITD for **5-3** is >5 while the value is higher than that of AC220 (2.5), suggesting that **5-3** exhibited greater selectivity to FLT3-ITD than AC220 (Figure 1D). CETSA indicated that **5-3** might directly bind to FLT3, improve its extracellular thermal stability in a dose-dependent manner, and protect it from temperature dependent degradation (Figure 1E). The synthesis, analysis of purity and NMR information of compound **5-3** are presented in Figures S1–S4.

### 2.2. **5-3** Significantly Suppressed FLT3 Signaling Pathway

**Figure 2:**
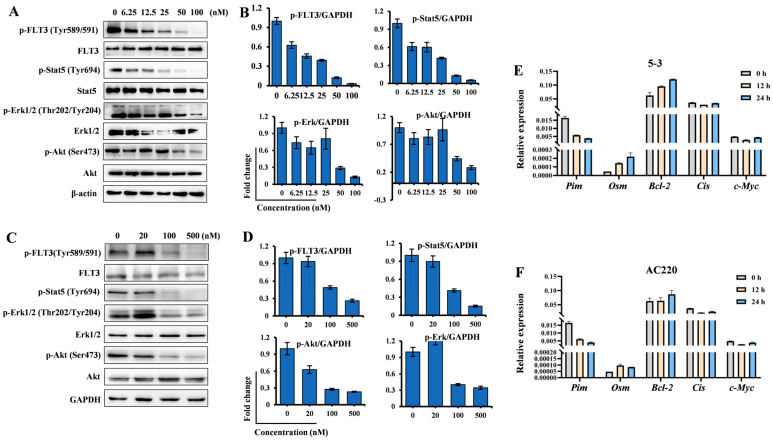
The impact of compound **5-3** on the FLT3 pathway in FLT3-ITD mutant cells. (**A**,**B**) The FLT3-ITD overexpressing BaF3 cells were treated with a range of concentrations (6.25, 12.5, 25, 50, and 100 nM) of compound **5-3** for 4 h. Subsequently, the whole-cell lysates were prepared and subjected to Western blot analysis to detect the phosphorylation levels of FLT3 and its downstream signaling molecules. (**C**,**D**) The FLT3-ITD mutant MV4-11 cells were treated with a series of concentrations (20, 100 and 500 nM) of compound **5-3** for 4 h. Subsequently, the proteins were extracted for Western blot analysis. (**E**,**F**) The FLT3-ITD mutant MV4-11 cells were treated with compound **5-3** (1000 nM) or AC220 (100 nM) for 12 and 24 h, respectively, and then the cDNA were extracted for RT-PCR analysis.

To confirm the attenuation of the FLT3 pathway in AML cells, we investigated the effects of compound **5-3** on the FLT3-mediated signaling pathway in BaF3-FLT3-ITD (Figure 2A,B) and MV4-11 (Figure 2C,D) cells. The phosphorylation levels of FLT3 and its downstream mediators, including STAT5, ERK, and AKT, were analyzed by Western blot. Compound **5-3** potently inhibited the phosphorylation of FLT3 and its downstream signaling proteins in a dose-dependent manner across both cell lines, thereby demonstrating its on-target effect. Notably, at a concentration of 100 nM, compound **5-3** almost completely blocked the phosphorylation of FLT3 (Tyr589/591), STAT5 (Tyr694), and ERK (Thr202/Tyr204) in both cell types. RT-PCR assay indicated that both **5-3** and AC220 decreased the level of Pim while no obvious change in the level of Bcl-2, Cis and c-Myc were observed (Figure 2E,F). Collectively, these findings indicate that compound **5-3** effectively interferes with the transduction of FLT3 signaling pathways in FLT3-ITD mutant cells.

### 2.3. Exerted Weaker Effect than AC220 on the Phosphorylation of c-KIT

**Figure 3:**
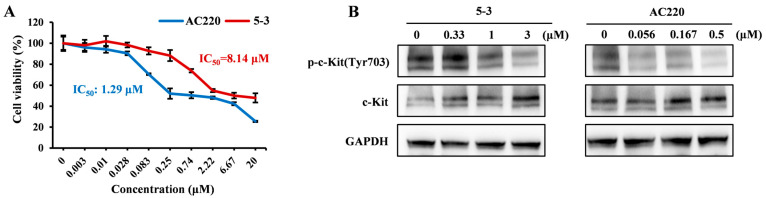
Effects of **5-3** on the phosphorylation of c-KIT. (**A**) The c-KIT overexpressing Kasumi-1 cells were treated with different concentrations of compound **5-3** and AC220 for 48 h, respectively. After treatment, cell viability was assessed by the MTT assay. (**B**) Kasumi-1 cells were treated with **5-3** and AC220 at different concentrations for 4 h, and then whole-cell lysates were subjected to Western blot analysis to detect the phosphorylation of c-KIT.

It has been reported that the myelosuppression toxicity associated with AC220 may potentially result from off-target effects, including the inhibition of c-KIT [20]. To explore the potential myelosuppression toxicity of compound **5-3**, we utilized Kasumi-1 cells, which exhibit high expression levels of the c-Kit protein, to evaluate the impact of **5-3** on c-Kit protein phosphorylation. The proliferation assay demonstrated that the IC_50_ values of compounds **5-3** and AC220 for Kasumi-1 cells were 8.14 μM and 1.29 μM, respectively (Figure 3A), indicating a ratio of approximately 6-fold. To investigate the effects of the compounds on c-Kit protein at an equivalent inhibitory level, in the subsequent Western blot analysis, the administration concentration of compound **5-3** was set to six-fold that of AC220. The WB results demonstrated that the inhibition of c-Kit phosphorylation by **5-3** was weaker compared to AC220. Therefore, it can be inferred that the potential toxicity of **5-3** toward c-Kit protein may be lower, and its safety profile may be more favorable than that of AC220 (Figure 3B).

### 2.4. **5-3** Specifically Targeting FLT3 Rather than β-Tubulin

**Figure 4:**
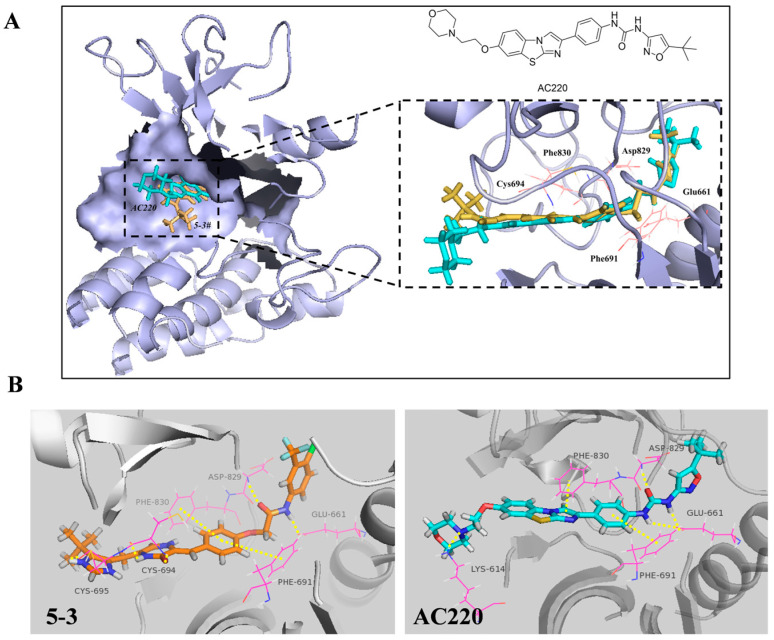
Binding mode of compound **5-3** within the crystal structure of FLT3 (PDB: 4XUF). (**A**) Compound **5-3** (orange sticks) and AC220 (blue sticks) are depicted along with the surface representation of the binding site. (**B**) The detailed view of the interactions is presented; side chains of residues involved in hydrophobic or π-interactions are depicted as gray sticks. Amino acid residues forming hydrogen bonds are illustrated with both main and side chains represented as cyan sticks. Other atoms are colored as follows: nitrogens (blue), oxygens (red), sulfur (yellow) and polar hydrogens (white).

Molecular docking showed that **5-3** fits the active pocket of FLT3, similar to AC220 (Figure 4A,B). The diketopiperazines of **5-3** forms hydrogen bonds with Cys694, the benzene ring engages in π-π interactions with Phe691 and Phe830, and the linker forms hydrogen bonds with Asp829 and Glu661. The docking score and the binding free energy of **5-3** are −14.144 and −100.17, respectively, which are better than that of the positive control AC220 (−13.258 and −106.333). In addition, the 50 ns molecular dynamics simulation of the FLT3/**5-3** complex, conducted using the Amber(Version 20) software package, revealed low root mean square deviation (RMSD) values throughout the trajectory (Figure S5). These observations indicate formation of a stable bound conformation between the FLT3 receptor and the **5-3**.

**Figure 5:**
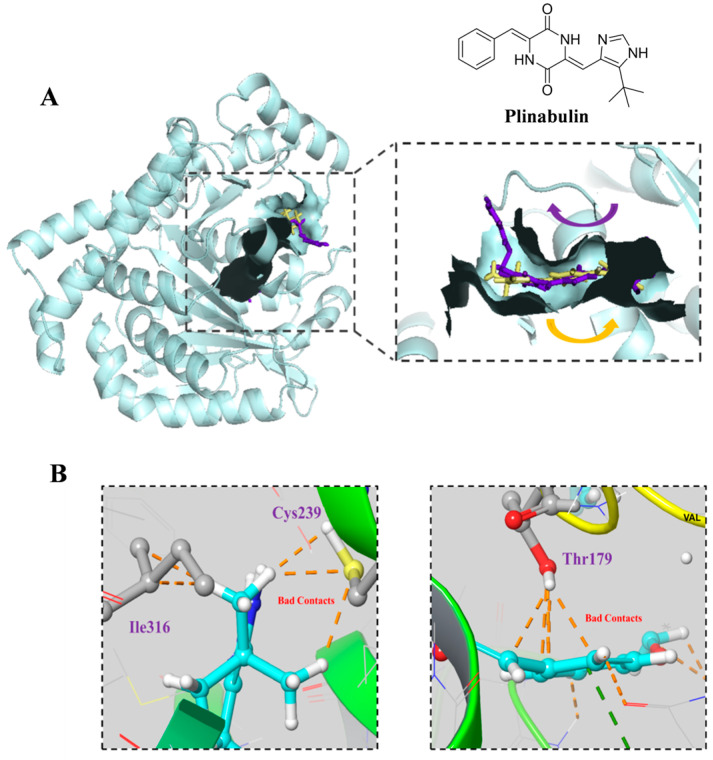
Interaction of compound **5-3** and Plinabulin with β-tubulin. (**A**,**B**) Binding modes of compound **5-3** and Plinabulin with β-tubulin (PDB: 5XHC), where the orange dotted lines represent unfavorable contacts.

As **5-3** was designed based on plinubulin, which inhibits cancer cell growth by targeting β-tubulin, we conducted molecular docking of **5-3** with β-tubulin (PDB: 4XHC) to determine if its potency in killing FLT3-ITD mutant cells is related to β-tubulin. The docking results indicated that plinabulin preferentially interacts with amino acid residues, including Cys239, Ile316, and Thr179, in β-tubulin. However, no favorable interactions were observed between these key amino acids and compound **5-3** (Figure 5A,B).

**Figure 6:**
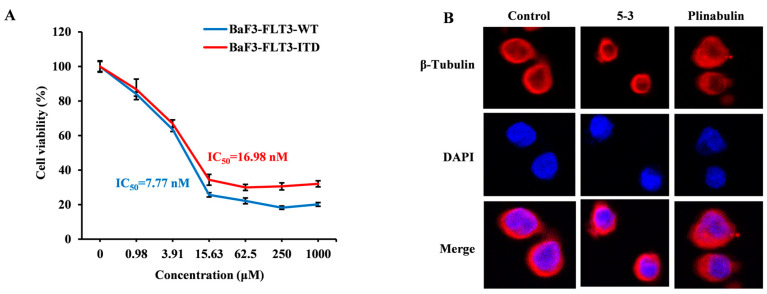
The activity of Plinabulin and **5-3** on β-tubulin. (**A**) The toxicity of Plinabulin on FLT3-ITD and FLT3-WT overexpressing BaF3 cells. (**B**) Immunofluorescence analysis of compound **5-3** and Plinabulin binding to β-tubulin. MV4-11 cells were treated with Plinabulin (5 nM) or compound **5-3** (200 nM) for 24 h, followed by staining with β-tubulin antibody and DAPI. Confocal microscopy was used to capture images, where red represents tubulin and blue represents DAPI.

The proliferation assay revealed that the IC_50_ values of plinubulin for FLT3-ITD and FLT3-WT overexpressing BaF3 cells were 16.98 nM and 7.77 nM, respectively, indicating no selective inhibition (Figure 6A). This is in stark contrast to compound **5-3**. Immunofluorescence analysis revealed that plinabulin induced clustering of microtubule proteins at 5 nM after 24 h of treatment. In contrast, the morphology of microtubule proteins treated with **5-3** at 200 nM was similar to that of the untreated group (Figure 6B).

### 2.5. **5-3** Induced FLT3-ITD Mutant AML Cells G1 Arrest a Dose-Dependent Manner

**Figure 7:**
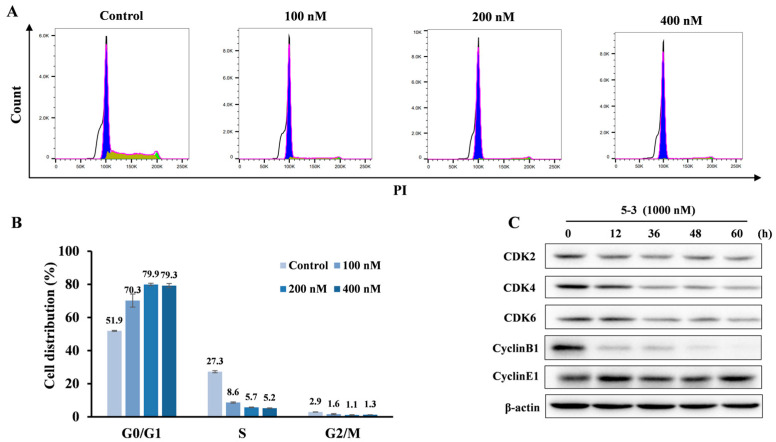
Effects of compound **5-3** on cell cycle distribution. (**A**) The original diagram illustrating the cell cycle arrest from flow cytometry. The blue, yellow and green colors represent the populations of G0/G1, S, and G2/M phase cells, respectively. (**B**). FLT3-ITD mutant MV4-11 cells were treated with compound **5-3** (at concentrations of 100, 200, and 400 nM) for 24 h, and cell cycle analyses were performed using DNA reagent kit by flow cytometry. (**C**). FLT3-ITD mutant MV4-11 cells were exposed to 1000 nM of compound **5-3** for 12, 36, 48 and 60 h, respectively, and subsequently subjected to SDS-PAGE for Western blot analysis.

To gain a further insight into the mechanism of cancer cell suppression activity, we examined cell cycle arrest of compound **5-3** against MV4-11 cells by flow cytometry. As shown in Figure 7A,B, **5-3** increased G1 population in a dose-dependent manner; for example, **5-3** resulted in 1.35, 1.53 and 1.53-fold increases in the percentage of G1 phase than the untreated group at the concentrations of 100, 200 and 400 nM, respectively. Western blotting assay indicated that **5-3** significantly induced the decrease in cell cycle-related proteins at 1000 nM, including CDK2, CDK4, CDK6, Cyclin B1 while no obvious change in Cyclin E1 and p21 (Figure 7C) was observed.

### 2.6. **5-3** Resulted Obvious Apoptosis in FLT3-ITD Mutant AML

**Figure 8:**
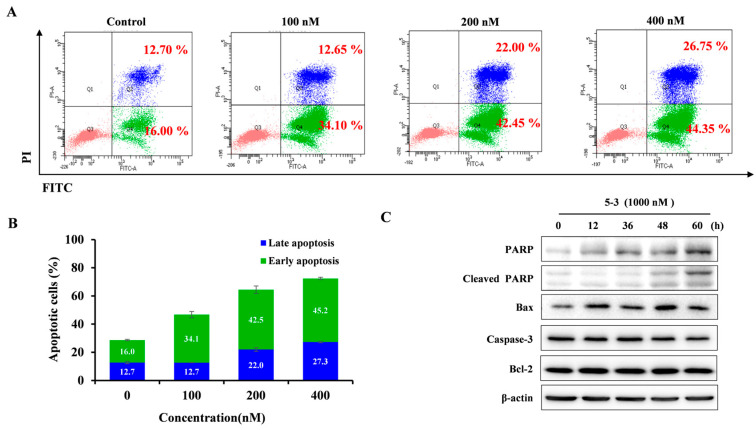
Effects of compound **5-3** on apoptosis. (**A**) The original diagram illustrating the apoptotic cells from flow cytometry. The red, blue, and green colors represent the populations of viable cells, early apoptotic cells, and late apoptotic cells, respectively (**B**) FLT3-ITD mutant MV4-11 cells were treated with compound **5-3** (at concentrations of 100, 200, and 400 nM) for 48 h, followed by flow cytometry analysis. (**C**) FLT3-ITD mutant MV4-11 cells were exposed to 1000 nM of compound **5-3** for 12, 36, 48 and 60 h, respectively, and subsequently subjected to SDS-PAGE for Western blot analysis.

We used FLT3-ITD mutant MV4-11 cells to evaluate the apoptotic effect of compound **5-3** on FLT3-dependent cells. Compared with the control group (28.7%), treatment with **5-3** resulted in 46.75, 64.45 and 72.40% apoptotic cells (both early and late apoptotic cells) at the concentration of 100, 200, and 400 nM, respectively (Figure 8A,B). The induction of apoptosis was further validated by subsequent Western blot analysis, which demonstrated that **5-3** increased the expression of cleaved poly (ADP-ribose) polymerase (PARP) and Bax in a dose-dependent manner in MV4-11 cells (Figure 8C).

## 3. Discussion

In this study, we identified diketopiperazines for the first time as a novel ATP-competitive moiety that can serve as a tyrosine kinase ligand. Additionally, by incorporating a hydrophobic region into the structure of Plinabulin, we developed a novel FLT3-ITD-selective inhibitor **5-3**. This inhibitor exhibits stronger selectivity for FLT3-ITD compared to AC220 while maintaining lower activity against FLT3-WT.

More importantly, the inhibitory effect of compound **5-3** on the proliferation of FLT3-ITD mutant cells is not associated with Plinabulin’s target, β-tubulin.

Type II FLT3 inhibitors typically bind to the DFG-out conformation (inactive form) of the FLT3 kinase domain. Generally, Type II FLT3 inhibitors exhibit enhanced selectivity compared to Type I inhibitors, owing to their additional interaction with the allosteric domain [21]. The development of FLT3-ITD inhibitors, such as AC220, represents a novel therapeutic strategy for acute myeloid leukemia (AML). However, despite the efficacy of FLT3 inhibition in both frontline and relapsed/refractory (R/R) settings, approximately 30%-45% of patients experience disease relapse following treatment. Additionally, side effects including asymptomatic QTc interval prolongation, febrile neutropenia, and pancytopenia, attributed to off-target effects, have been documented [22]. Our research focuses on identifying more potent, selective, and safer FLT3-ITD inhibitors for the clinical management of FLT3-ITD + positive AML.

Based on the publication, the motif of type II FLT3 inhibitor generally consists of three key regions: the solvent region, the hinge region and the hydrophobic region. The hydrophobic interaction region typically involves five/six-membered heterocyclic ring, such as those found in AC220, Clifutinib, CHMFL-FLT3-213 [23,24,25,26]. The solvent region is commonly characterized by a morpholine ring [23,25,26,27] and piperazine ring [28,29], such as AC220, KX2-391 and LT-850-166. However, innovation predominantly occurs in the hinge region. For instance, replacing benzo[d]pyrrolo[2,1-b]thiazole in AC220 with phenylacetylene led to the development of Clifutinib [24], which is currently in Phase III clinical trials. Similarly, substituting benzo[d]pyrrolo[2,1-b]thiazole in AC220 with pyrazolo [3,4-d]pyrimidine resulted in the discovery of CHMFL-FLT3-213 [25]. These findings suggest that the hinge region plays a determinant role in the design of type II FLT3 inhibitors. Therefore, widening the scope of novel hinge region modifications could provide new opportunities for drug discovery.

Diketopiperazines, which possess two hydrogen bond donors and two hydrogen bond acceptors, enable these compounds to exhibit strong biological and pharmacological activities. As a result, diketopiperazines have emerged as an important pharmacophore in medicinal chemistry. However, plinabulin remains the only anti-cancer candidate featuring a diketopiperazine moiety. Plinabulin (BPI-2358) is a synthetic analog of phenylahistin, a natural product isolated from Aspergillus species. It is classified as a colchicine-site microtubule destabilizing agent [30].

Patrick J. Cimino et al. reported that Plinabulin inhibits the growth of KRAS-driven cancer cells and improves survival in a KRAS-driven mouse gene transfer glioma model [31]. James R. Tonra et al. reported that Plinabulin alleviates neutropenia induced by microtubule-stabilizing, DNA cross-linking, and DNA intercalating chemotherapies without affecting bone marrow or blood G-CSF levels [32].

## 4. Materials and Methods

### 4.1. Compounds, Cell Lines and Plasmids

All cell lines used were obtained from American Type Culture Collection (Manassas, VA, USA) and incubatedat 37 °C in a humidified atmosphere (CO_2_ 5%–95% air). The human FLT3-ITD mutant leukemia cells (MV4-11 and MOLM-13) and FLT3 wide-type leukemia cells (HL-60) were all cultured in IMDM medium supplemented with 20% FBS (ExCell). The murine BaF3 leukemia cells were cultured in IMDM medium. The human colon cancer HCT116 cells were cultured in McCoy’s 5A medium containing 10% FBS. The human breast cancer MDA-MB-231 cells were cultured in Leibovitz’s L-15supplemented with 10% FBS. The human embryonic kidney HEK293 cells were cultured in DMEM supplemented with 10% FBS. The human acute myeloblastic leukemia Kasumi-1 cells were cultured in RPMI-1640 medium supplemented with 20% FBS. All type of the cell culture mediums were purchased from Thermo Fisher(Grand Island, New York, USA).All the human cells were verified by STR detection. The concentration of penicillin-streptomycin (HyClone) for the mentioned cells were all 1%. The cells were passaged every 2 to 3 days and used within 20 passages. Plasmids for FLT3-wt and FLT3-ITD were purchased from Genomeditech company (Shanghai, China). The FLT3-wt and FLT3-ITD overexpressing BaF3 cells were constructed by Lentivirus transfection. AC220 were purchased from Aladdin Scientific (ShangHai, China). Plinabulin was synthesized by our group. The purity values were both more than 99%.

### 4.2. Cytotoxicity Assay

An amount of 100 μL of the cell suspension was seeded into a 96-well plate, with a cell density of 1.5 × 10^4^ cells per well. 100 μL medium containing the detected compounds was furtheradded to co-incubate for another 48 h at 37 °C with 5% CO_2_. MTT (Sigma-Aldrich, St. Louis, MO, USA) solution (5 mg/mL) was subsequently added to each well to react for an additional 6 h in the incubator. Absorbance values at a wavelength of 490 or 570 nm were measured using SPECTRA max i3 microplate reader (Molecular Devices, San Jose, CA, USA). Inhibitory concentration (IC_50_) values were calculated using nonlinear best fit regression analysis by Prism 8.0. The experiments were repeated for three times.

### 4.3. Immunofluorescence

The ability of compound **5-3** and plinabulin to disrupt microtubules was evaluated as previously described [33]. After the treatment of **5-3** or plinabulin, MV4-11 cells were fixed with pre-warmed 4% paraformaldehyde at room temperature for 20 min. Following centrifugation, 1 × 10^6^ cells were resuspended in 0.1 mL of PBS, and 10 μL of the suspension was deposited onto a glass slide. Subsequently, the samples were blocked with 3% BSA in 0.5% Triton X-100 for 20 min at 25 °C. Microtubules were detected using a primary antibody against β-tubulin and a Cy3-conjugated secondary antibody. Nuclei were stained with DAPI. Coverslips were analyzed by confocal microscopy (Leica TCS-SP8 SR, Wetzlar, Germany) at a magnification of 630×. The immunofluorescence optical density (IOD) of β-tubulin (red fluorescence) was quantified using Image-Pro Plus 6.0 (Media Cybernetics, Inc., Rockville, MD, USA). The experiments were repeated three times.

### 4.4. Western Blotting Analysis

The cells were treated with compound **5-3** (at concentrations of 6.25, 12.5, 25, 50, and 100 nM) for 4 h. Total protein was extracted using lysis buffer (Cell Signaling Technology, Danvers, MA, USA) and quantified using a BCA kit (Solarbio, Beijing, China). The whole-cell lysates were then subjected to SDS-PAGE for separation and subsequently transferred onto a 0.22 μm pore-sized nitrocellulose membrane. After transfer, the membrane was blocked with 5% skimmed milk powder. The membrane was incubated overnight at 4 °C with primary antibodies against FLT3 pathway proteins. These primary antibodies were purchased from Cell Signaling Technology (Danvers, MA, USA). The proteins were detected using the Tanon imaging analysis system (Tanon, Shanghai, China). The experiments were repeated for three times.

### 4.5. Molecular Docking

The protonated 3D was employed using standard bond lengths and angles, using Molecular Operating Environment (MOE) software (Version 2020). Then, the geometry optimization and energy minimization were applied to obtain the Conf Search module in MOE, followed by saving the MOE file for the upcoming docking process. The X-ray crystallographic structure of FLT3 (PDB: 4XUF) and β-Tubulin (PDB: 5XHC) was retrieved from the protein data bank at a resolution of 2.75 Å.

### 4.6. Molecular Dynamics Simulation

The molecular system was meticulously constructed using the xL EaP module within the AMBER (Version 20) software package. Protein and peptide components were parameterized with the ff19SB force field, while the General Amber Force Field (GAFF) was specifically employed for ligand. The system was subsequently solvated in a truncated octahedral box of TIP3P water molecules, maintaining a minimum 10 Å buffer distance between the solute and box boundaries in AMBER22. System neutrality was achieved through the addition of sodium counterions, followed by a comprehensive energy minimization protocol. The initial minimization phase involved constraining the solute with a harmonic force constant of 10 kcal·mol^−1^·Å^−2^, utilizing 3000 steps of steepest descent followed by 3000 steps of conjugate gradient minimization. This was succeeded by an unconstrained minimization to ensure system stability. For system equilibration, the temperature was gradually increased from 50 to 300 K over a 100 ps simulation in the NVT ensemble, with solute positions restrained using a harmonic force constant of 5 mol^−1^·Å^−2^. Finally, production molecular dynamics (MD) simulations were performed in the NPT ensemble, during which positional restraints were removed to allow for dynamics.

### 4.7. ADP-Glo Assay

Active FLT3-WT and FLT3-ITD enzyme were purchased from Signal Chem (Richmond, BC, Canada).Kinase was mixed with a 5-fold inhibitor and pre-incubated for the time indicated at each assay (5–30 min). Kinase reaction buffer A (4 μL) and a mixture (4 μL) of myelobasic protein (final concentration 0.1 mg/mL) and ATP (final concentration 100 μM to 1 mM) was added, and the kinase reaction was incubated at room temperature for 2 h. ADP-Glo reagent (10 μL) was added and incubated for 40 min at room temperature. Then, the kinase detection reagent (20 μL) was added and incubated for 30 min (100 μM ATP) to 60 min (1 mM ATP) at room temperature. Relative inhibition was calculated compared to vehicle control and reagent control and were plotted as relative inhibition of kinase activity to the logarithmic concentration of the inhibitor using Prism 8.0 (GraphPad, San Diego, CA, USA). The experiments were repeated for three times.

### 4.8. Cell Thermodynamic Stability Analysis (CETSA)

MV4-11 cells and **5-3** were co-incubated for 3 h at the incubator, followed by centrifugation at 1000 rpm for 5 min. MV4-11 cells were re-suspended in PBS buffer containing 1% protease inhibitor and phosphatase inhibitor, and then repeatedly frozen and thawed for three times in liquid nitrogen. The proteins were then heated by gradient Bio-rad PCR instrument (Heracles, CA, USA) for 5 min, and subjected to centrifugation to collect the supernatant. The expression level changes in the FLT3 protein were analyzed via Western blot. The proteins were detected using Tanon imaging analysis system (Shanghai, China). The experiments were repeated for three times.

### 4.9. Cell Apoptosis Detection

MV4-11 cells (3 × 10^5^ cells per well) were seeded into a 6-wells for 24 h, and were treated with difererent concentrations of compound **5-3** for 48 h. All cells (including dead cells) were collected, then centrifuged at 800 rpm for 3 min, and washed with PBS two times. 1 × 10^6^ cells were resuspended in 1× binding buffer, and were incubated with 5 μL of FITC-conjugated annexin V and 10 μL of propidium iodide (PI) for 5 min at room temperature in the dark before detection. The samples were analyzed by BD FACSAriaIII flow cytometry (Franklin Lakes, NJ, USA). The experiments were repeated for three times.

### 4.10. Cell Cycle Detection

Assessment of cell cycle distribution was carried out by using a cell cycle staining kit (MULTISCIENCES, Hangzhou, China) according to the manufacturer’s instructions and analyzed by flow cytometry (BD FACSAriaIII, Franklin Lakes, NJ, USA). MV4-11 cells (3 × 10^5^ cells per well) were seeded into a 6-wells for 24 h, and were treated with difererent concentrations of compound **5-3** for 24 h. The cells collected, then centrifuged at 800rpm for 3 min, and washed with PBS. 10 μL of PI was added according to the kit, and samples were incubated for 30 min at room temperature in the dark. The samples were analyzed by flow cytometry (BD FACSAriaIII, Franklin Lakes, NJ, USA). The data was analyzed using FLOWJO V10.6.2 software (BD, Franklin Lakes, NJ, USA). The experiments were repeated for three times.

### 4.11. Real Time PCR

MV4-11 cells were treated with **5-3** at concentrations of 1000 nM for 0, 5 and 10 h, respectively. RNA extraction was performed using a chloroform extraction method. The concentration of RNA was determined using a Nanodrop spectrophotometer (Wilminton, DE, USA) and diluted to less than 1000 ng with an appropriate volume of diethyl pyrocarbonate (DEPC) water if necessary. cDNA synthesis was carried out utilizing reverse transcription kits(Yisheng, Shanghai, China). The expression levels of indicated genes were quantified through Roche RT-PCR equipement (Basel, Switzerland) analysis. The experiments were repeated for three times. Primers used for PCR amplification are listed as follows:
*Cis*Forward primer 5′-GCATAGCCAAGACCTTCTCCTAC-3′
Reverse primer 5′-ACGTGCCTTCTGGCATCTTCTG-3′*Bcl-2*Forward primer 5′-CTTTGAGTTCGGTGGGGTCA-3′
Reverse primer 5′-GGGCCGTACAGTTCCACAAA-3′*Pim*Forward primer 5′-TCTACTCAGGCATCCGCGTCTC-3′
Reverse primer 5′-CTTCAGCAGGACCACTTCCATG-3′*Osm*Forward primer 5′-CACACAGAAACCCCAGTCCCA-3′
Reverse primer 5′-GACACCATCGTTCCCGTCCTA-3′*c-Myc*Forward primer 5′-CACTAACATCCCACGCTCTGA-3′
Reverse primer 5′-AAATCATCGCAGGCGGAACA-3′*GAPDH*Forward primer 5′-ACAACTTTGGTATCGTGGAAGG-3′
Reverse primer 5′-GCCATCACGCCACAGTTTC-3′

### 4.12. Statistical Analysis

No statistical methods were used to predetermine the sample size. Data were plotted using GraphPad Prism v8.0 software as mean values, with error bars indicating standard deviation. * *p* < 0.05, ** *p* < 0.01 and *** *p* < 0.001, respectively, unless otherwise specified.

## 5. Conclusions

The study identified diketopiperazines for the first time as a novel ATP-competitive moiety serving as a tyrosine kinase ligand. Additionally, by incorporating a hydrophobic region into plinabulin’s structure, we developed a novel FLT3-ITD-selective inhibitor **5-3**, which may serve as a drug candidate for the treatment of FLT3-ITD-positive AML.

## Data Availability

The original contributions presented in this study are included in the article and Supplementary Materials. Further inquiries can be directed to the corresponding authors.

## Supplementary Materials

The following supporting information can be downloaded at: https://www.mdpi.com/article/10.3390/md23070289/s1, Figure S1. The synthesis scheme of **5-3**; Figure S2. The H NMR spectra of **5-3**; Figure S3. The C NMR spectra of **5-3**; Figure S4. Analysis of **5-3** purity by HPLC; Figure S5. Evolution of the root mean square deviation (RMSD) for complex of **5-3** and FLT3 in the 50 ns molecular dynamics (MD) simulations.

## Abbreviations

AML	Acute myeloid leukemia
FLT3	Fms-liketyrosine receptor kinase 3
ITD	internal tandemduplication
TKD	Tyrosine kinase domain
CETSA	Cellular thermal shift assay
DKPs	Diketopiperazines
AC220	Quizartinib
PI	Propidium iodide
IOD	Immunofluorescence optical density
GAFF	General amber force field
MD	Molecular dynamics
DEPC	Diethyl pyrocarbonate
RMSD	Root mean square deviation
