# Supplementary material for: The Novel Diketopiperazine Derivative, Compound 5-3, Selectively Inhibited the Proliferation of FLT3-ITD Mutant Acute Myeloid Leukemia (AML) Cells
Source: Mar Drugs. 2025 Jul 16;23(7):289. doi: 10.3390/md23070289 (PMC12299508; doi:10.3390/md23070289)
Supplement: Supplementary file 1 [file marinedrugs-23-00289-s001.zip › marinedrugs-3719795-supplementary.pdf]

**1. Method and Reagents**

Chemical design and synthesis materials

All chemicals and solvents were purchased from commercial sources and used without further purification. Reactions were monitored through thin-layer chromatog-raphy (TLC) and visualized under UV light, or phosphomolybdic acid solutions as a developing stain. Flash column chromatography was performed with silica gel(200–300 mesh). 1H NMR and 13C NMR spectra were recorded either on a 400 MHz JEOL Instrument at 25 ◦C or 600 MHz Bruker Instrument at 25 ◦C, using TMS as an internal standard, respectively.

To a solution of 2-(4-formylphenoxy)acetic acid (90.0 mg, 0.50 mmol) in DCM (3mL) was added 4-chloro-3-(trifluoromethyl)aniline (107.3 mg, 0.55 mmol), EDCI(105.4 mg, 0.55 mmol )and HOBT(74.3mg, 0.55 mmol). Subsequently the reaction mixture was stirred at room temperature for 12 h . When there was no starting material remained by TLC analysis, extract the mixture three times with dichloromethane, water, and saturated sodium chloride solution, and concentrated under reduced pressure . The residue was purified by silica gel flash column chromatography (PE:EA=5:1-3:1) to provide N-(4-chloro-3-(trifluoromethyl)phenyl) -2-(4-formylphenoxy)acetamidein a yield 50% .

To a solution of glycine anhydride (50 g，438 mmol) in acetic anhydride. (179 g，1753 mmol). Subsequently the reaction mixture was stirred at 150 ℃ for 30 h then the mixture was concentrated under reduced pressure . The crude product was triturated with ethyl acetate to afford 1,4-diacetylpiperazine-2,5-dione in a yield 85% as brown solid.

To a solution of 5-(tert-butyl)-1H-imidazole-4-carbaldehyde (6 g, 39.20 mmol) in DMF (40 mL) was added 1,4-diacetylpiperazine-2,5-dione (14 g, 0.58 mmol),$Cs_2CO_3$ (19 g, 58.8 mmol). Subsequently the reaction mixture was stirred at 50 ℃ for 12 h under $N_2$. When there was no starting material remained by TLC analysis, the reaction solution was poured into cold water, filtered, and concentrated under reduced pressure. The residue was purified by silica gel flash column chromatography (PE:EA=2:1-1:1) to provide (Z)-1-acetyl-3-((5-(tert-butyl)-1H-imidazol-4-yl)methylene)piperazine-2,5-dione in a yield 52% as brown solid.

To a solution of (Z)-1-acetyl-3-((5-(tert-butyl)-1H-imidazol-4-yl)methylene) piperazine-2,5-dione (87.1 mg, 0.3 mmol) in DMF (5 mL) was added N-(4-chloro-3-(trifluoromethyl)phenyl)-2-(4-formylphenoxy)acetamide (107.1 mg, 0.3 mmol),$Cs_2CO_3$ (117.3 mg, 0.36 mmol). Subsequently the reaction mixture was stirred at 50 ℃ for 12 h under $N_2$. When there was no starting material remained by TLC analysis, the reaction solution was poured into cold water， filtered, and concentrated under reduced pressure. The residue was purified by silica gel flash

column chromatography (PE:EA=2:1-1:1) to provide compound **5-3** in a yield 57% as yellow solid.

The synthesis scheme of 5-3 is shown in Supplementary figure 1.

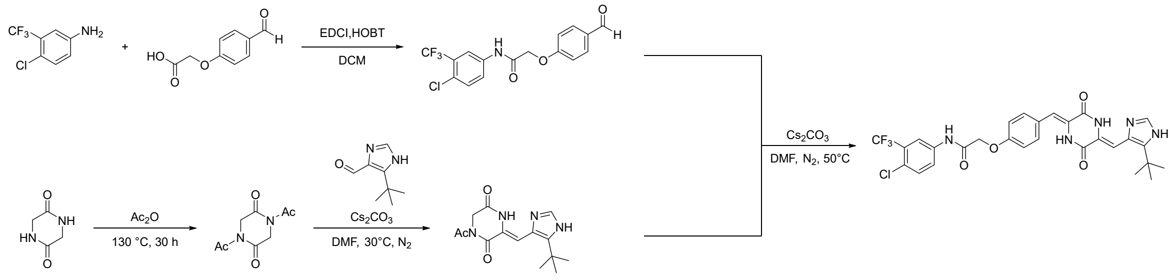


Supplementary Figure S1. The synthesis scheme of 5-3.

**The purity detection of 5-3**

Analysis of compound 5-3 purity by HPLC system (Waters e2695, USA) using a reversed-phase C18 column. HPLC conditions are as follows: solvent A, Methanol; solvent B, $H_2O$; flow rate of 1.0 mL/min, from 40% B to 0% B in 15 min, from 0% B to 0% B between 15 min and 20 min, from 0% B to 40% B between 20 min and 25 min. UV detection was at 365 nm.

2. Results

Chemistry

2-(4-((Z)-((Z)-5-((5-(tert-butyl)-1H-imidazol-4-yl)methylene)-3,6-dioxopiperazin-2-ylidene)methyl)phenoxy)-N-(4-chloro-3-(trifluoromethyl)phenyl)acetamide (**5-3**)

$^1$H NMR (400 MHz, DMSO-$d_6$) δ 12.28 (s, 1H), 12.17 (s, 1H), 10.54 (s, 1H), 9.96 (s, 1H), 8.21 (d, *J* = 2.6 Hz, 1H), 7.91 (dd, *J* = 8.8, 2.6 Hz, 1H), 7.81 (s, 1H), 7.66 (d, *J* = 8.8 Hz, 1H), 7.49 (d, *J* = 8.9 Hz, 2H), 7.02 (d, *J* = 8.8 Hz, 2H), 6.80 (s, 1H), 6.68 (s, 1H), 4.76 (s, 2H), 1.34 (s, 9H).( Supplementary Figure S2)

$^{13}$C NMR (101 MHz, DMSO-$d_6$) δ 167.76, 158.11, 158.00, 157.08, 140.74, 138.41, 134.83, 132.66, 131.45, 131.30, 126.99, 125.77, 124.98, 124.40, 118.99, 118.88, 115.49, 114.43, 105.39, 67.52, 32.44, 31.17, 29.58. (Supplementary Figure S3)

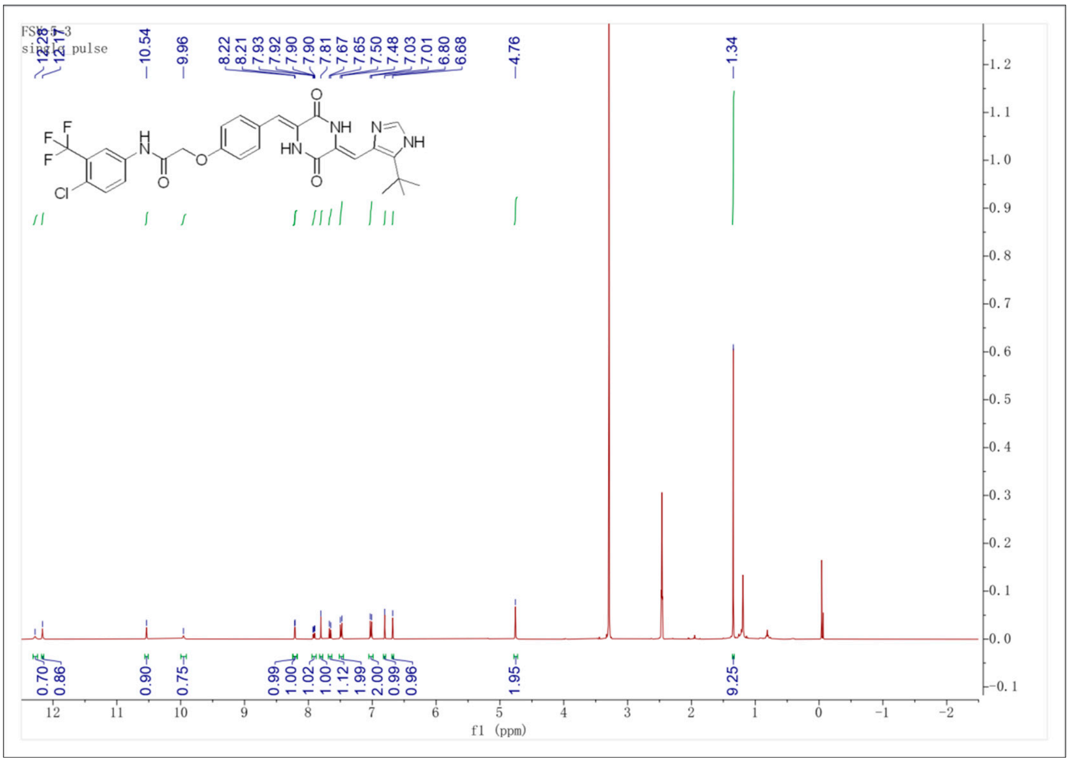


Supplementary Figure S2. The H NMR spectra of 5-3.

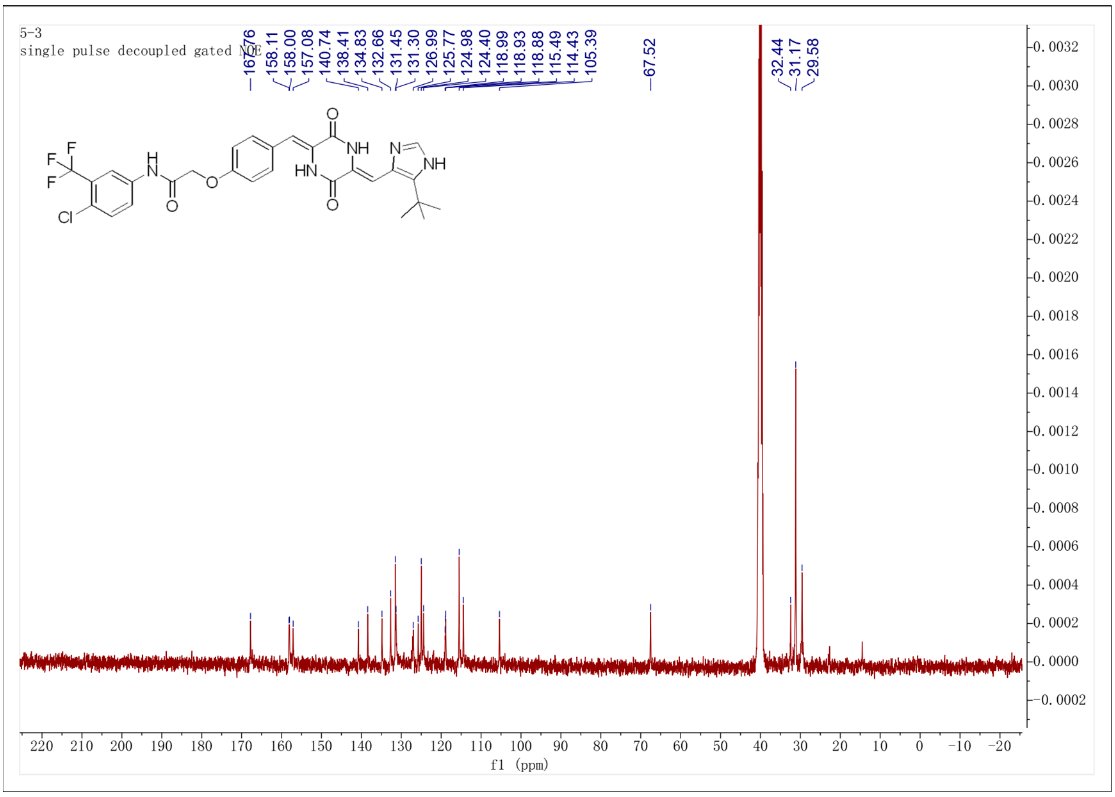


Supplementary Figure S3. The C NMR spectra of 5-3.

The purity of 5-3

The purity of the compound is more than 95% as determined by integrating the peak area (Supplementary figure 4)

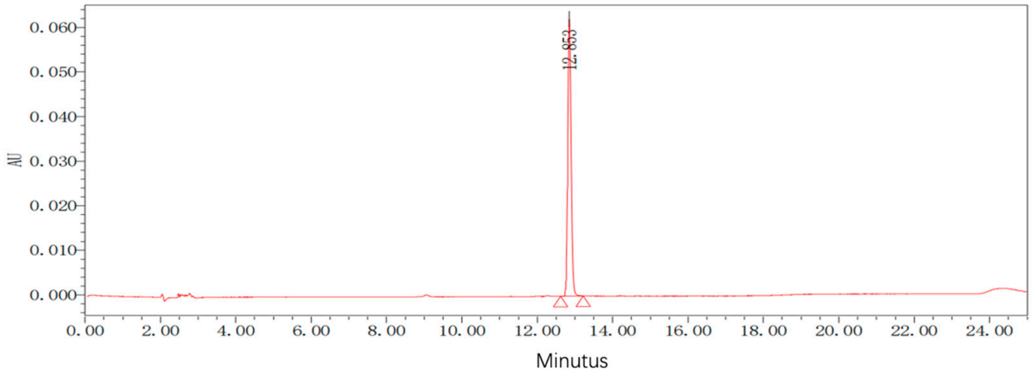


Supplementary Figure S4. Analysis of 5-3 purity by HPLC.

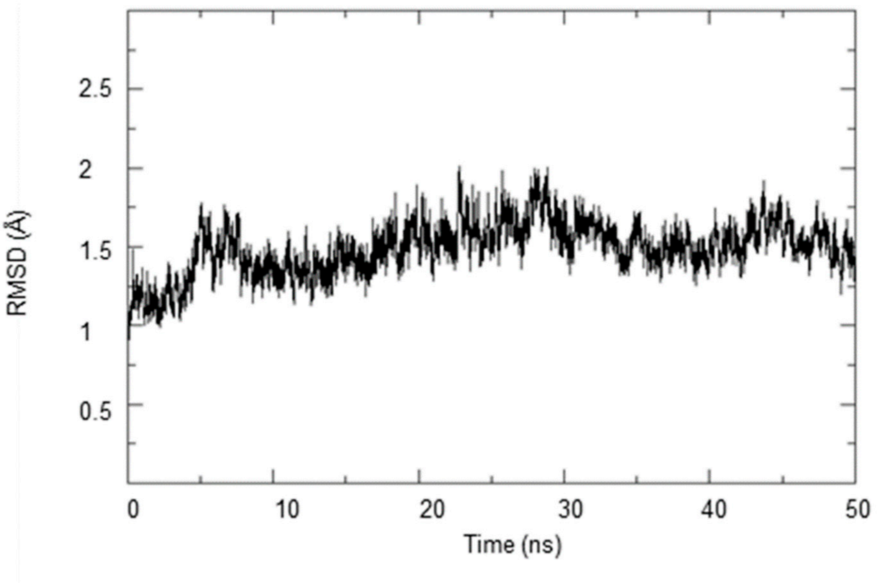


Supplementary Figure S5 Evolution of the root mean square deviation (RMSD) for complex of 5-3 and FLT3 in the 50 ns molecular dynamics (MD) simulations.
